# In Vitro Characterization of Adipose Stem Cells Non-Enzymatically Extracted from the Thigh and Abdomen

**DOI:** 10.3390/ijms21093081

**Published:** 2020-04-27

**Authors:** Elena Dai Prè, Alice Busato, Silvia Mannucci, Federica Vurro, Francesco De Francesco, Valentina Riccio, Samantha Solito, Reetuparna Biswas, Paolo Bernardi, Michele Riccio, Andrea Sbarbati

**Affiliations:** 1 Department of Neuroscience, Biomedicine and Movement, Human Anatomy and Histology Section, University of Verona, 37129 Verona, Italy; elena.daipre@univr.it (E.D.P.); alice.busato@univr.it (A.B.); silvia.mannucci@univr.it (S.M.); federica.vurro@univr.it (F.V.); reetuparna.biswas@univr.it (R.B.); paolo.bernardi@univr.it (P.B.); andrea.sbarbati@univr.it (A.S.); 2 Department of Computer Sciences, University of Verona, 37135 Verona, Italy; 3 Accademia del Lipofilling, Research and Training Center in Regenerative Surgery, 61025 Montelabbate (PU), Italy; michele.riccio@ospedaliriuniti.marche.it; 4 Department of Reconstructive Surgery and Hand Surgery, AOU “Ospedali Riuniti”, 60020 Ancona, Italy; 5 School of Biosciences and Veterinary Medicine, University of Camerino, 62032 Matelica, MC, Italy; valy.riccio91@gmail.com; 6 Centro Piattaforme Tecnologiche, University of Verona, 37135 Verona, Italy; samantha.solito@univr.it

**Keywords:** adipose stem cells, adipose tissue, non-enzymatic method, Rigenera^®^ protocol, abdomen, thigh

## Abstract

Autologous fat grafting is a surgical technique in which adipose tissue is transferred from one area of the body to another, in order to reconstruct or regenerate damaged or injured tissues. Before reinjection, adipose tissue needs to be purified from blood and cellular debris to avoid inflammation and preserve the graft viability. To perform this purification, different enzymatic and mechanical methods can be used. In this study, we characterized in vitro the product of a closed automatic device based on mechanical disaggregation, named Rigenera^®^, focusing on two sites of adipose tissue harvesting. At first, we optimized the Rigenera^®^ operating timing, demonstrating that 60 s of treatment allows a higher cellular yield, in terms of the cell number and growth rate. This result optimizes the mechanical disaggregation and it can increase the clinical efficiency of the final product. When comparing the extracted adipose samples from the thigh and abdomen, our results showed that the thigh provides a higher number of mesenchymal-like cells, with a faster replication rate and a higher ability to form colonies. We can conclude that by collecting adipose tissue from the thigh and treating it with the Rigenera^®^ device for 60 s, it is possible to obtain the most efficient product.

## 1. Introduction

Whilst adipose tissue has been considered a waste product for years, its essential role as a regenerative agent has only recently been recognized [1,2]. Indeed, autologous fat grafting has many applications, including breast reconstruction following tumor therapies [3,4], the treatment of burn scars and congenital and post-traumatic malformations [5,6], and rejuvenation aims [7]. It is believed that the main agents responsible for regeneration are adipose-derived stem cells (ASCs), which are adult plastic-adherent mesenchymal stem cells abundant in, and easily isolable from, adipose tissue [8,9,10]. They self-renew and are multi-potent, which means that they have the ability to differentiate, under appropriate stimulation, into different mesodermal cell lineages, especially into adipocytes, osteocytes, and chondrocytes [11,12,13,14,15,16]. After liposuction, the processing of adipose tissue is a key step in avoiding inflammation and preserving the fat graft viability. The traditional purification method is enzymatic digestion, which involves the use of collagenase. Briefly, this method consists of collagenase digestion, centrifugation, and washing steps to remove red blood cells, allowing adipocyte separation from dense cellular tissue, called the stromal-vascular fraction (SVF) [17]. The SVF contains different kinds of cells, including ASCs, mesenchymal and endothelial progenitor cells, leukocytes, and pericytes. Although the enzymatic method is the most effective for SVF isolation, it is an expensive and time-consuming open system, which requires a further step of enzyme purification. In addition, it destroys the stem-cell niche, known as the microenvironment, which surrounds the stem cell, allowing interactions with neighboring cells that promote cell survival, proliferation, and differentiation. Moreover, according to the Good Manufacturing Practice regulations of the European Parliament and Council (EC regulation no. 1394/2007), only minimal cell manipulation is allowed in a clinical setting. Therefore, enzymatic methods are definitely forbidden [18,19]. In clinics, the necessity to purify the adipose tissue in the operating room immediately after the surgery without laboratory processing and in sterile conditions is mandatory. For these reasons, many companies have developed automatic closed devices based on mechanical methods [20,21,22,23,24,25]. One of these, named Rigenera^®^ (HBW, Turin, Italy), composed of an engine and disposable sterile capsules, uses mechanical disaggregation operated by steel blades rotating at 80 rpm, followed by filtration through 70–80 µm pores, to produce immediately injectable micrografts.

The present study aimed to in vitro characterize the product obtained by the Rigenera^®^ device in terms of the cell viability and morphology, immunophenotyping, colony-formed unit, and differentiation potential. The study focused on adipose tissue isolated from the thigh and abdomen.

## 2. Results

**Figure 1:**
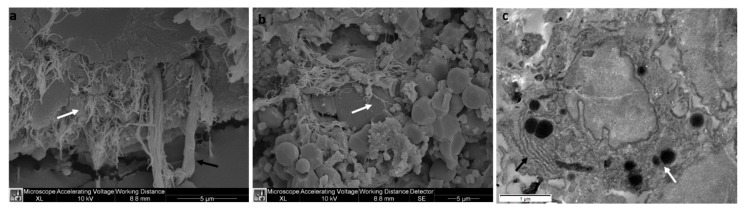
Representative SEM (**a**,**b**) and TEM (**c**) images of the Rigenera^®^ product obtained from the thigh. The Rigenera^®^ device provides fragments consisting of elastic fibers ((**a**) black arrow), collagen fibers ((**a**,**b**) white arrow), and different kinds of isolated cells (**b**), including mesenchymal-like cells (**c**), characterized by rough endoplasmic reticulum (black arrow) and lipidic droplets (white arrow). No differences at an ultrastructural level were found between the two sites of adipose tissue collection (thigh and abdomen).

The Rigenera^®^ device is a technology which allows the mechanical disaggregation of a small amount of adipose tissue, in order to obtain an autologous product that is able to promote regeneration. The procedure consists of tissue manipulation conducted by a ceramic blade and filtration (filter of 80 μm). After processing, the product is collected from the reservoir located at the bottom of the removable capsule of the Rigenera^®^ device. At first, the composition of the product obtained from the mechanical disaggregation and harvested from two different anatomical sites—the thigh and abdomen—was examined at an ultrastructural level. In Figure 1, SEM and TEM images of the product obtained from adipose tissue of the thigh are shown. No difference was observed at an ultrastructural level between the two sites of adipose tissue collection (images not shown). The adipose tissue SEM images (Figure 1a,b) show connective tissue fragments consisting of elastic fibers (see the black arrow in Figure 1a), collagen fibers (white arrow in Figure 1a,b), and different kinds of isolated cells (Figure 1b). Among the isolated cells, mesenchymal-like cells (Figure 1c), characterized by rough endoplasmic reticulum (see the black arrow in Figure 1c) and small size lipidic droplets (white arrow, Figure 1c) were visualized by TEM. 

**Figure 2:**
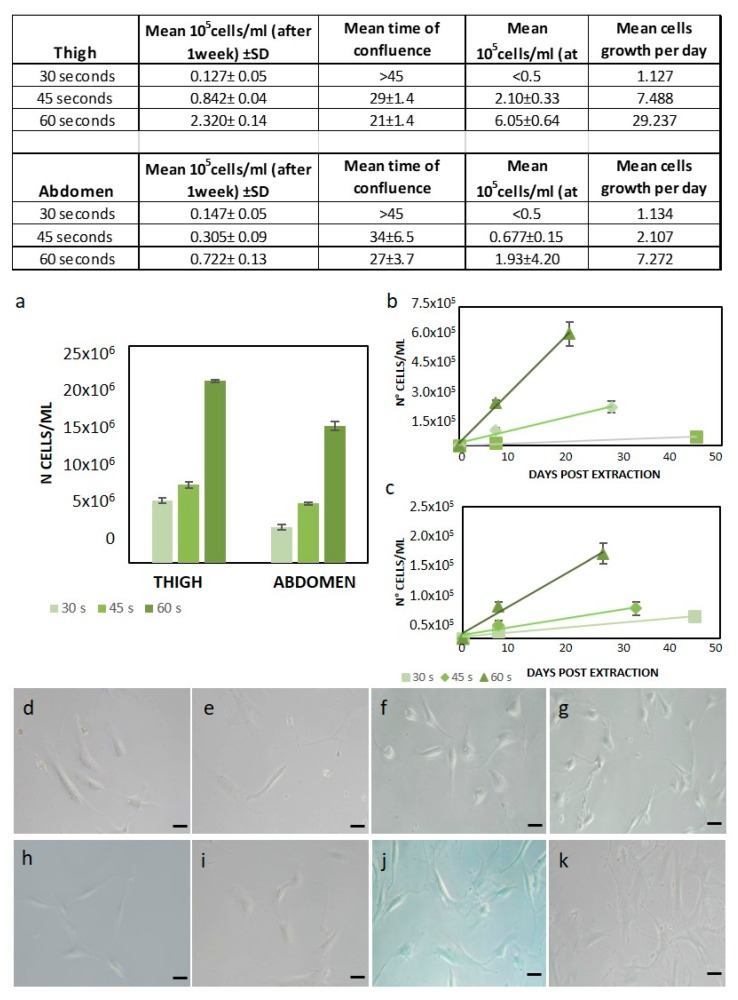
Rigenera^®^ method optimization. The tabs show all of the results obtained. (**a**) Cell viability test with the trypan blue exclusion method. At passage 0, the number of total cells was much higher with the 60 s Rigenera^®^ treatment compared to the other timings (30 and 45 s). After one week, the number of pure adipose-derived stem cells (ASCs) was still higher with the 60 s Rigenera^®^ treatment in both the thigh (**b**) and abdomen (**c**). Microscopic images (resolution 10×) of cells extracted from the thigh (**d**–**f**) and abdomen (**h**–**k**) with the Rigenera^®^ device operating at different timings of 30 s (**d**,**h**), 45 s (**e**,**i**), and 60 s (**f**,**j**), and the enzymatic method (**g**,**k**).

In order to determine how the mechanical processing time (seconds of sample treatment with ceramic blades) affects the cellular yield of the device, the number of ASCs, the growth rate, and the cell morphology were evaluated. Based on preliminary results [18], the best processing time was chosen among 30, 45, and 60 s. At first, the cells were counted at passage 0 (Figure 2A). The number of cells with 60 s treatment was much higher compared to 45 s and 30 s (respectively, 15.16 × 10^6^ ± 0.49 compared to 6.84 × 10^6^ ± 0.19 and 4.13 × 10^6^ ± 0.33 for the abdomen and 21 × 10^6^ ± 0.16 compared to 9 × 10^6^ ± 0.35 and 7.2 × 10^6^ ± 0.28 for the thigh, as shown in Figure 2a). After one week, only cells capable of forming fibroblast-like colonies attached to the flask and were countable. Compared to the 60 s treatment, the thigh cell yield after the 45 s treatment was about 42% and after 30 s, it was about 20% (see the tab in Figure 2). In addition, the mean time of confluence was 8 ± 2.8 days lower with 60 s treatment than with 45 s treatment. The 30 s-treated cells grew very slowly and were discarded after 45 days (Figure 2b). In the case of abdominal cell extraction, the cell yield of the 60 s treatment was about 36% compared to the 45 s treatment, whereas the 30 s-treated cell yield was about 5%. The mean time of confluence was 7 days less with 60 s treatment than with 45 s treatment (Figure 2c). Additionally, for abdomen samples, the 30 s treatment cells grew very slowly and were discarded after 45 days (see the tab in Figure 2). By calculating the mean cell growth per day, 60 s treatments showed a higher replication rate (29.23 × 10^3^ and 7.27 × 10^3^ for the thigh and abdomen, respectively), as reported in the table of Figure 2. The ASC micrographs in Figure 2 (Figure 2d–k) clearly represent the cell morphology in the flask. The Rigenera^®^ treatment did not affect the cell morphology compared to cells obtained from enzymatic digestion (g–k), exhibiting a homogeneous fibroblast-like morphology. Indeed, no signal of suffering was observed and the membranes and nuclei were well-preserved (Figure 2d–f,h–j for the thigh and abdomen, respectively). By analyzing the results obtained for the cell yield, number of cells, and growth rate, the 60 s treatment can be considered the most efficient for the Rigenera^®^ method.

**Figure 3:**
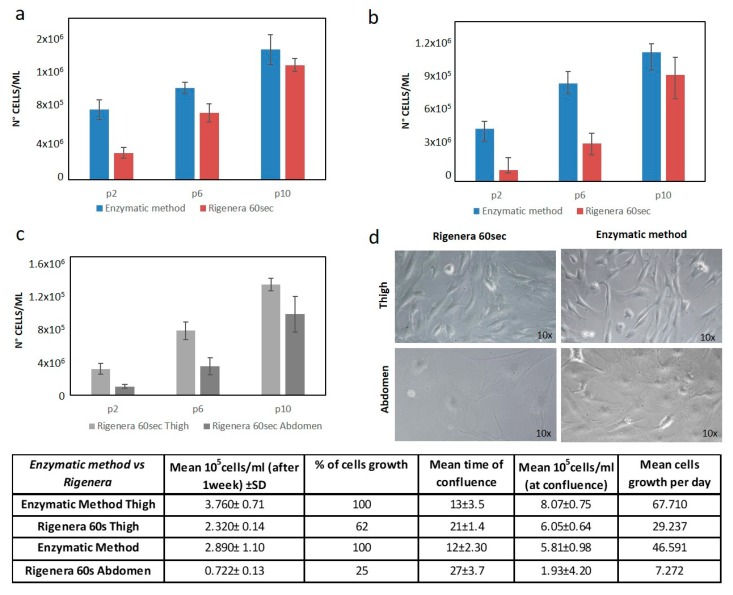
Comparison of Rigenera^®^ and the enzymatic method. (**a**) Growth rate comparison of ASCs from Rigenera^®^ extracted from the thigh and the enzymatic method. (**b**) Growth rate comparison of ASCs from Rigenera^®^ extracted from the abdomen and the enzymatic method. (**c**) Growth rate comparison of ASCs extracted with Rigenera^®^ from the thigh and abdomen. (**d**) Microscopic images (resolution 10×) of ASCs at high passages (p10). The tab shows all of the results obtained.

Next, the cells obtained from 60 s treatment were compared with those obtained with the enzymatic method. After one week of culture, the Rigenera^®^ cell yield for the thigh was 62% and for the abdomen, was around 25%, compared to the enzymatic method (see the tab in Figure 3). 

**Figure 4:**
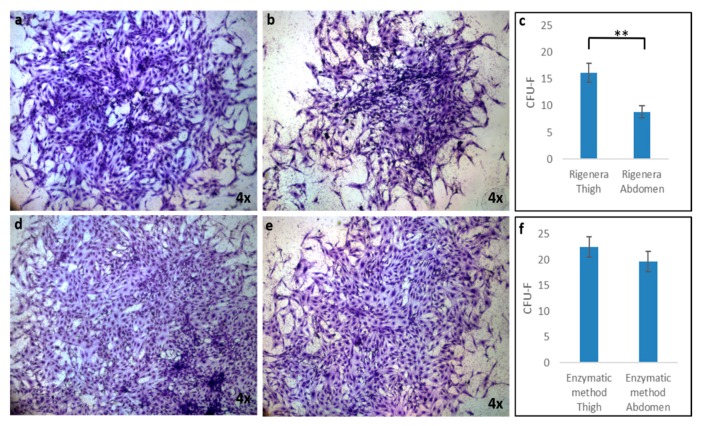
The colony-forming unit-fibroblast (CFU-F) assay of ASCs obtained by Rigenera^®^ ((**a**) thigh and (**b**) abdomen) and the enzymatic method ((**d**) thigh and (**e**) abdomen). CFU-F values were determined at day 15 after plating using the Toluidine Blue staining method. ASCs extracted from the thigh (**a**–**d**) formed larger colonies containing more cells than those extracted from the abdomen (**b**–**e**); (**c**) CFU-F numbers at day 15 obtained by the Rigenera^®^ device; CFU-F numbers showed significant differences between the two groups (thigh and abdomen). (**f**) CFU-F numbers at day 15 obtained by the enzymatic method; CFU-F numbers showed no significant differences between the two groups (thigh and abdomen). Data are presented as the mean ± SE, with *n* = 6 (** *p* < 0.01).

Additionally, the replication rate was lower when using the Rigenera^®^ device: extracted cells reached the confluence in 21 ± 1.4 and 27 ± 3.7 days with Rigenera^®^ (for the thigh and abdomen, respectively), while, when using the enzymatic method, they took 13 ± 3.5 and 12 ± 2.30 days (for the thigh and abdomen, respectively), as reported in the tab of Figure 4.

Comparing the two sites of extraction, the cell yield for the abdomen was about 31% lower than that of the thigh. Moreover, the cells extracted from the thigh reached confluence 6 ± 2.3 days before the cells extracted from the abdomen (see the tab in Figure 4), demonstrating a higher replicative rate. 

The histograms in Figure 3a,b show the number of cells from cellular passages 2 (p2), 6 (p6), and 10 (p10). Although the resulting rate of replication was higher with the enzymatic method, and the cells obtained with the enzymatic method were able to reach confluence faster than cells obtained with the Rigenera^®^ method, at high passages (i.e., 10), no statistically significant difference in cell number was observed between the Rigenera^®^-obtained cells and Collagenase digestion (Figure 3a,b, *p*-value < 0.05). This means that, at these passages, the growth rate was comparable. Figure 3c compares the replication rate (in terms of the number of cells at passage 2–6 and 10) of the thigh and abdomen. The difference is clear at the low cellular passage (3.02 × 10^5^ cells for the thigh and 9.92 × 10^4^ cells of the abdomen at passage 2), while the difference between the thigh and abdomen was not statistically significant after a long period of culture and many passages (such as at p10) (*p*-value < 0.05) (Figure 3c). Finally, the morphological analysis highlighted a slight difference between the thigh and abdomen: for example, the cells obtained from the abdomen were flatter and more widely spread (see Figure 3d).

In order to compare the ability to form colonies of ASCs obtained from the thigh and abdomen, colony-forming unit-fibroblast (CFU-F) assays were performed. Figure 4 displays representative micrographs of CFU-F detected by Toluidine Blue staining after 15 days of Rigenera^®^ treatment (Figure 4a, thigh, and b, abdomen) compared with enzymatic digestion (Figure 4d, thigh, and b, abdomen). The images show that both ASCs treated with Rigenera^®^ and isolated from the thigh and abdomen were able to grow forming clusters, but larger colonies (formed by a higher number of cells) could be observed in samples obtained from the thigh compared to those from the abdomen (Figure 4a,b). These differences are not evident in the samples treated with enzymatic digestion (Figure 4d,e). Moreover, when the CFU-F numbers were counted, more colonies were detected in samples isolated from the thigh (16.17 ± 1.8) compared to the abdomen (8.83 ± 1.1), as reported in Figure 4c. No statistical differences in the number of CFU-F between thigh and abdomen samples treated with enzymatic digestion were found.

**Figure 5:**
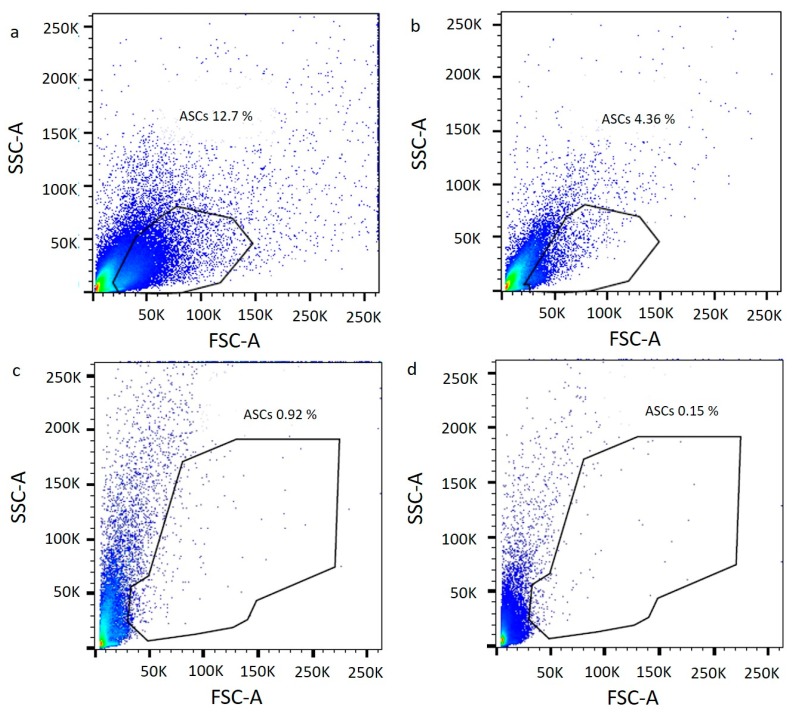
Cytograms of enzymatic digestion (**a**, thigh; **b**, abdomen) and Rigenera^®^ product (**c**, thigh; **d**, abdomen) at passage 0. Mesenchymal-like cells was selected based on Forward Scatter (FSC) and Side Scatter (SSC) information.

In order to demonstrate the presence of ASCs in the Rigenera^®^ product, an immunophenotypic assay at p0 (immediately after the treatments) was performed. Figure 5 shows a scatter plot that combines the signals obtained from the Forward Scatter (FSC) and Side Scatter (SSC). Based on the size, shape, and internal structure of cells, it was possible to select the mesenchymal-like cells presented in the scatter plot. The cytogram at p0 confirmed the much higher yield of stem cells for the enzymatic method (12.7% of ASCs from the thigh and 4.36% of ASCs from the abdomen) (Figure 5a for the thigh, and b for the abdomen) compared to Rigenera^®^ (0.92% of ASCs from the thigh and 0.15% of ASCs from the abdomen) (Figure 5c for the thigh, and d for the abdomen). 

**Figure 6:**
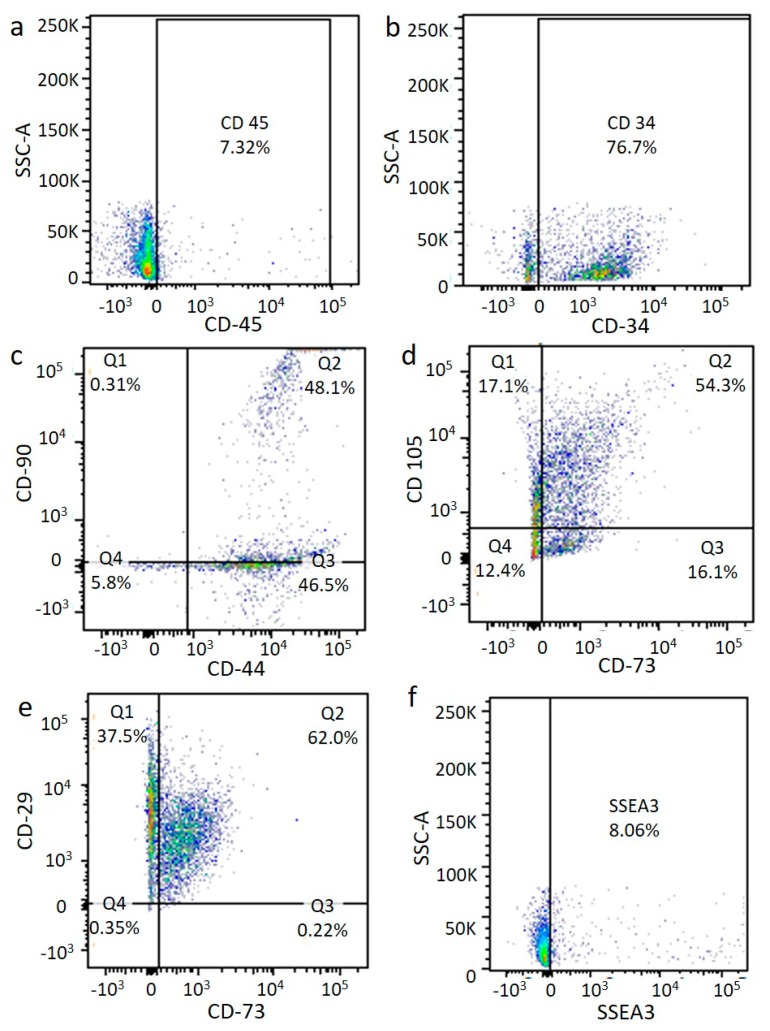
Flow cytometry after enzymatic digestion. Immunophenotyping analysis of ASCs at *p*0 from enzymatic digestion. Cell markers CD45 (**a**), CD34 (**b**), CD44/CD90 (**c**), CD73/CD105 (**d**), CD73/CD29 (**e**), and SSEA3 (**f**).

Subsequently, specific single antigens or a combination of two antigens were tested on the previously selected cells. At p0, the ASCs isolated by the enzymatic method, including CD105, CD90, CD73, CD44, and CD29 (Figure 6c–e), were expressed at a medium level, while the hematopoietic marker CD45 (Figure 6a) was poorly expressed and the hematopoietic marker CD34 was highly expressed (Figure 6).

**Figure 7:**
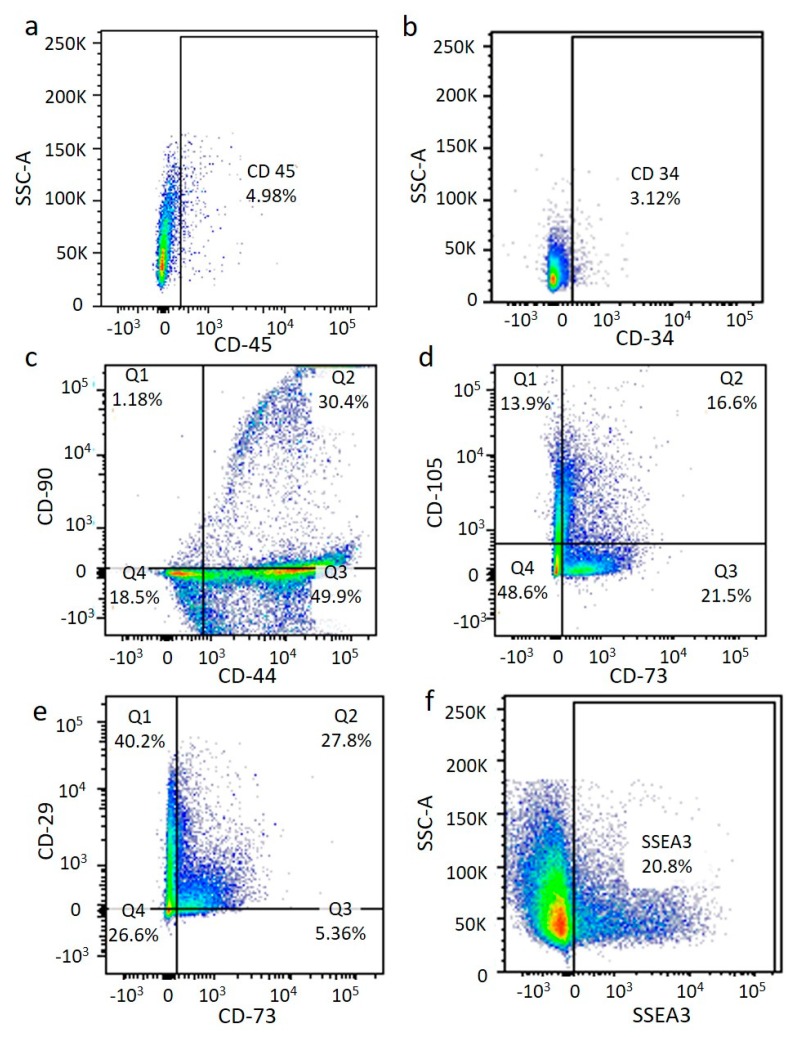
Flow cytometry after Rigenera^®^ treatment. Immunophenotyping analysis of ASCs at *p*0 from Rigenera^®^ treatment. The presence of the ASC phenotype is confirmed. Cell markers CD45 (**a**), CD34 (**b**), CD44/CD90 (**c**), CD73/CD105 (**d**), CD73/CD29 (**e**), and SSEA3 (**f**).

An immunophenotyping analysis of the ASCs obtained from the Rigenera^®^ method at p0 showed a medium expression of mesenchymal stem cell markers (CD105, CD73, and CD29) (Figure 6c–e) comparable with the results of the enzymatic method, confirming the presence of the ASC phenotype. In contrast, a different expression of the hematopoietic marker CD34 (Figure 6b) was observed for the two techniques. Indeed, this antigen was more highly expressed after collagenase digestion, probably meaning that the Rigenera^®^ method allowed the isolation of a purer cell population (see Figure 7). Finally, we also identified a generally low (8%) and medium (20.8%) presence of multi-lineage differentiating stress enduring cells (MUSE cells) for the enzymatic and Rigenera^®^ method, respectively (see Figure 6f and Figure 7f). No differences in the marker expression level were found for the thigh and abdomen (data not shown). 

At a higher passage (p10), the antigen pattern was similar between cells obtained from the Rigenera^®^ and enzymatic method, resulting in a high expression of the mesenchymal stem cell surface marker and confirming phenotype maintenance. The immunophenotypic analysis at p10 also showed that the surface marker expression profiles of ASCs from the thigh and abdomen were comparable and preserved over time. 

**Figure 8:**
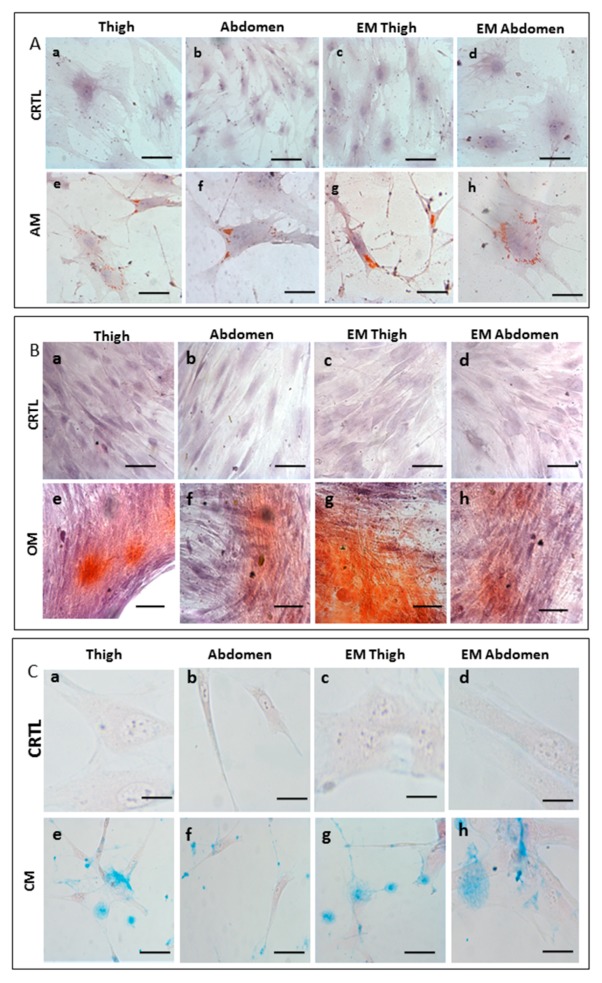
Cell differentiation assay. Multilineage differentiation potential of the ASCs from the thigh and abdomen after treatment with Rigenera^®^ compared to enzymatic methods (EM). (**A**) Cells were cultured with complete ASC medium (CRTL) (**a**,**b**,**c**, scale bar 100 μm, **d** scale bar 50 μm) and adipogenic medium (AM) (**e**,**f**,**g** scale bar 100 μm, **h** scale bar 50 μm). In the adipogenic medium, adipogenesis was indicated by the accumulation of neutral lipid vacuoles stained with Oil Red O, while the nucleus was stained with hematoxylin. (**B**) Cells were cultured with complete ASC medium (CRTL) (**a**,**b**,**c** scale bar 100 μm, **d** scale bar 50 μm) and osteogenic medium (OM) (**e**,**f**,**g**,**h** scale bar 100 μm). In osteogenic medium, osteogenesis was indicated by Alizarin Red S staining of extracellular matrix calcification, while the nucleus was stained with hematoxylin. (**C**) Cells were cultured with complete ASC medium (CRTL) (**a**,**c**,**d** scale bar 50 μm, **b** scale bar 100 μm) and chondrogenic medium (CM) (**e**,**f**,**g** scale bar 100 μm, **h** scale bar 50 μm). In chondrogenic medium, chondrogenesis was shown by the deposition of sulfated proteoglycan-rich matrix stained with Alcian blue, while the nucleus was stained with hematoxylin.

In order to determine the multipotency of ASCs isolated from samples treated with Rigenera^®^, a differentiation assay for adipocyte, chondrocytes, and osteocytes was performed. In this experiment, we observed cellular differentiation macroscopically and the results were compared with those of the enzymatic method and non-induced cells (Figure 8). The adipogenic differentiation of ASCs was confirmed by Oil Red O staining after 14 days of induction. Compared to the non-induced cells (Figure 8A (a–d)) the cells stained with Oil Red O solution showed the formation of lipid droplets within the cytoplasm (Figure 8A (e–h)). Moreover, there were no notable differences between ASCs isolated from the thigh and abdomen. The osteogenic potential was determined by Alizarin Red Staining (Figure 8B (e–h)) to indicate the extracellular matrix calcification. Both samples (thigh and abdomen) demonstrated positive staining in osteo-induced cells in contrast to the non-induced cells, indicating the osteogenic differentiation of ASCs. Finally, ASCs were chondrogenically cultured for 3 weeks and stained with Alcian blue. Chondrogenesis was observed in all samples by the deposition of sulfated proteoglycan-rich matrix (Figure 8C (e–h)). The differentiation potential between cells obtained with Rigenera^®^ and enzymatic digestion was comparable. This result shows that ASCs obtained with Rigenera^®^ treatment are able to differentiate towards multilineage cell fates.

## 3. Discussion

Although the enzymatic method, which has been used for 40 years in the laboratory in order to isolate cells, is the best method available, it is definitely not compatible with clinics, due to the long-lasting procedure and legal restrictions [19,26,27,28,29,30,31,32,33]. Furthermore, it destroys the stem-cell niche, which is the microenvironment which surrounds the stem cell, allowing interactions with neighboring cells and promoting cell survival, proliferation, and differentiation [34,35]. Many efforts have been made to establish a mechanical method with a yield comparable to the one of collagenase. Unfortunately, so far, none of them have exhibited the same performance. In addition, in order to use it in clinics, the method has to be fast, safe, standardized, and autologous. Rigenera^®^ addresses all of these requirements. This technology allows the mechanical disaggregation of a small amount of adipose tissue previously harvested from the same patient. Rigenera^®^ technology provides selective filtration, applying a filter of 80 μm, and the product is collected from the reservoir located at the bottom of the Rigenera^®^ device, making it a safe and closed device. The Rigenera^®^ device can be used for various problems, such as ulcers, alopecia, and post traumatic skin defects [36,37,38,39,40,41].

The product obtained with this technology consists of fragments of connective tissue (collagen and elastic fibers) and isolated cells (Figure 1), among which mesenchymal-like cells, which are considered the main agent responsible for the regenerative potential of the product [8,9]. In this study, we conducted characterization in terms of the cell viability, cell morphology, and immunophenotyping the mesenchymal-like cells obtained with the Rigenera^®^ device.

We optimized the Rigenera^®^ operating time, demonstrating that, to obtain a higher yield of cells, the best choice is the mechanical disaggregation of adipose tissue for 60 s. Indeed, by processing the adipose tissue by the ceramic blades contained in Rigenera^®^ for 60 s, we obtained a higher number of cells with better a replication capacity compared to the 30 and 45 s treatment. Our hypothesis is that a short sample processing time is not enough to produce the proper mechanical disaggregation of fat tissue, allowing only a partial extraction of cells contained within it. These limitations could affect the clinical product’s efficiency.

We also proved that Rigenera^®^ treatment does not affect the cell morphology, since the cell appearance under the microscope was not altered and was also preserved over time (passages higher than the ninth). In addition, when plated in a flask, the cells obtained by the Rigenera^®^ device were able to grow forming clusters, which is a typical feature of mesenchymal cells, named the colony-forming capacity. Moreover, the differentiation potential of cells extracted with the Rigenera^®^ device towards adipogenic, osteogenic, and chondrogenic lineages was demonstrated. Unfortunately, the cell yield and thus, the mean time of confluence, was lower compared to that of the enzymatic method, but the replication rate was comparable at higher passages (from the tenth passage). The antibody expression of the typical mesenchymal stem cell markers (CD105, CD90, CD73, CD44, and CD29) and the hematopoietic markers (CD45 and CD34) was similar to that of the collagenase method and preserved over time. Therefore, no alteration in the ASC phenotype was observed. Moreover, an average expression of the MUSE-SSEA3 antigen was detected. MUSE cells are a subpopulation of mesenchymal stem cells, double-positive for the mesenchymal marker CD105 and the pluripotency marker stage-specific embryonic antigen-3 (SSEA-3), discovered by Kuroda in 2010 [12]. They are known to be pluripotent, i.e., they are able to differentiate into cells belonging to the three germ layers and able to endure stress, such as oxygen deprivation. For these reasons, they could be considered the main agent responsible for regeneration and reparation [12,13]. The presence of MUSE cells, together with multipotent cells, allows a significant advantage in tissue regeneration, as MUSE cells are present in a variety of connective tissues. This provides safety and ethical advantages for clinical applications.

Finally, since surgeons harvest adipose tissues from various anatomical districts [42], we compared the regenerative potential of the cells obtained from the thigh and abdomen. Although subcutaneous regions are considered the most appropriate sites to harvest adipose tissue [42,43], we demonstrated that the ASC yield (in terms of the number of cells extracted, replicative rate, and number of CFU) from the thigh was higher than that from the abdomen, using the Rigenera^®^ device. We found that the progenitor cell frequencies (the ability to form colonies) were higher in ASCs extracted from the thigh: these ASCs could produce more colonies containing larger numbers of cells compared to those from the abdomen. Moreover, the immunophenotypic analysis confirmed that a much higher yield of ASCs was obtained from thigh samples, although both the samples (thigh and abdomen) expressed a phenotype profile specific for ASC markers. Moreover, a difference between cells obtained from the thigh and abdomen was appreciable in terms of the cell morphology: abdomen cells were flatter and more widely spread in the flask. This large and flat cell morphology is reflected in the lower replication rate (number of cells at confluence) and in the greater amount of time required to reach confluence compared to cells extracted from the thigh. These results demonstrated the characteristics of senescent cells [44]. These differences could be due to the fact that the thigh is often a virgin site of extraction and the adipose tissue remains more uniform, with a collagen matrix being thinner than adipose tissue obtained from the abdomen, resulting in a tissue that is easier to process with the Rigenera^®^ device. 

## 4. Materials and Methods

### 4.1. Adipose Tissue Sample Collection

The current non-enzymatic system (named Rigenera^®^, HBW, Turin, Italy) was designed to collect and prepare human disaggregated biological tissue, such as dental pulp [25], dermis [26], scars [27], cartilage [19], and adipose tissue, for re-injection. In this study, adipose tissue was harvested from nine women subjected to liposuction, aged between 41 and 69 years. Consent was obtained prior to tissue collection, according to the ethical guidelines set by the review board for human studies. The tissue was subsequently processed with Rigenera^®^ technology with 16 mL capsules, as described previously by De Francesco and colleagues [18].

### 4.2. Cell Isolation and Culture

Each adipose tissue sample was divided into two portions. In the Rigenera^®^ capsule, 4 mL of lipoaspirate and 4 mL of the complete culture medium Dulbecco Minimum Essential Medium (DMEM) (Merck KGaA, Darmstadt, Germany) containing 10% Fetal Bovine Serum (FBS), 1% of a mix of penicillin/streptomycin 1:1 (GIBCO Life Technology, Monza, Italy), and 0.5% amphotericin B (GIBCO Life Technology, Monza, Italy) were added. The Rigenera^®^ device was operated for 30, 45, or 60 s. The collected cell pellet was withdrawn from the capsule by a syringe, filtered through a 70-µm nylon mesh, and centrifuged at 3000 rpm for 7 min. The supernatant was discarded, and the cell pellet was resuspended in 6 mL of complete medium, plated in a 25 cm^2^ flask (BD FalconTM, Becton Dickinson, Milano, Italy), and incubated at 37 °C and 5% CO2. The second portion of lipoaspirate was digested with collagenase following the collagenase protocol, as reported in [23]. Briefly, 4 mL was digested with 1 mg/mL type I collagenase (GIBCO life technology, Monza, Italy) in Hank’s Balanced Salt Solution (HBSS) and 2% bovine serum albumin (BSA) at 37 °C for 45 min. The enzymatic action was neutralized by adding complete medium. Then, the sample was centrifuged at 3000 rpm for 7 min, the supernatant was discarded, and the cell pellet was incubated with 3 mL of 160 mM NH4Cl at room temperature for 10 min to lyse the erythrocytes. After centrifugation, the cells were resuspended in 6 mL of complete medium, filtered through a 70-µm nylon mesh, plated in a 25 cm^2^ flask with complete culture medium, and incubated at 37 °C and 5% CO2. The medium was first changed after 72 h and, successively, every 48 h. At confluence, cells were detached by incubating them with trypsin-EDTA 1% (GIBCO Life Technology, Monza, Italy) at 37 °C for 5 min and re-plated in a 75 cm^2^ flask.

### 4.3. Morphological Analysis: Scanning and Transmission Electron Microscopy 

For transmission electron microscopy, the pellets of the product obtained after the treatment with Rigenera 60’’ were fixed with glutaraldehyde 2% in Sorensen buffer pH 7.4 for 2 h, post-fixed in 1% osmium tetroxide in aqueous solution for 2 h, and dehydrated in graded concentrations of acetone. At the end of the dehydrating process, samples were positioned in a multi-well grid for electron microscopy and observed using an EM10 electron microscope (Zeiss, Oberkochen, Germany).

For scanning electron microscopy analysis, the pellets of the product obtained after the treatment with Rigenera 60” were fixed to 2% glutaraldehyde in a phosphate buffer for 2–4 h, post-fixed in 1% osmium tetroxide in the same buffer for 1 h, and dehydrated in graduated acetone concentrations. The samples were then treated with a critical-point dryer (CPD 030, Balzers Vaduz, Liechtenstein), mounted on metal samples, and coated with gold (MED 010 Balzers). SEM imaging was performed with XL30 ESEM (FEI-Philips Eindhoven, The Netherlands).

### 4.4. Morphological Analysis and Cell Viability Test

Morphological analysis of the cells obtained with the three different Rigenera^®^ operating timings and the enzymatic method, and from the two different harvesting sites, was performed by visualizing them under a light microscope (Optika Microscopes, Ponteranica, Italy). The cell viability and growth rate were evaluated by the cell viability test with the trypan blue exclusion method.

### 4.5. Cell Colony-Forming Unit Assay

A colony-forming unit-fibroblast (CFU-F) assay was performed for cells treated with Rigenera^®^ and enzymatic digestion. Briefly, cells isolated from the thigh and abdomen and treated with the Rigenera^®^ device were plated in 6-well culture plates at a density of 3000 cells/cm^2^ and cultured in the complete media. On the 15th day after plating, the total number of cell colonies (CFU-F, a cluster of at least 50 adhered and fibroblast-like cells) was stained with Toluidine Blue (Sigma) and counted. 

### 4.6. Immunophenotyping

After isolation, cells were counted and 2 × 10^5^ cells were placed in a tube for cytofluorimetric analysis. The pellet was washed with 1 mL of 1% FBS in PBS and then labeled with fluorescent-dye conjugated antibodies in a final volume of 100 µL and incubated for 30 min in ice. The examined antibodies were APC-conjugated CD90 (dilution 1:5), PerCP-Cyt5.5-conjugated CD105 (dilution 1:20), BV421-conjugated CD73 (dilution 1:20), BV785-conjugated CD44 (dilution 1:20), PE-conjugated CD34 (dilution1:5), FITC-conjugated CD29 (dilution 1:20), and BV650-conjugated CD45 (dilution 1:20). All of the antibodies were purchased from BD Biosciences, (Becton Dickinson Italy S.p.A., Milan, Italy). Alexa Fluor-488-conjugated SEEA3 (dilution 1:20) was purchased from Aurogene (Aurogene S.R.L, Rome, Italy). After the incubation, the pellet was rinsed, resuspended in 300 µL of 1% FBS in PBS, and transferred in flow cytometry tubes. The immunophenotyping was performed through an FACS canto II (BD, Becton Dickinson, Italy).

### 4.7. Cell Differentiation Assay

The multilinear differentiation potential was evaluated by testing the ability of the product obtained after treatment with Rigenera (60 s) and the enzymatic method to differentiate into adipocytes, chondrocytes, and osteocytes. Briefly, adipocyte differentiation was achieved after 16 days culture of MSCs with adipogenic medium, containing 10^−6^ M dexamethasone, 10 μg/mL insulin, and 100 μg/mL 3-isobutyl-1-methylxantine (Sigma). Chondrocyte differentiation was achieved after 14 days culture with the StemPro osteogenesis differentiation kit (GIBCO Life Technology, Italy). Osteoblast differentiation was achieved after 21 days culture with the StemPro osteogenesis differentiation kit (GIBCO Life Technology, Italy). Oil Red O, Alcian blue, and Alizarin Red Stain were employed to identify adipocytes, chondrocytes, and osteocytes, respectively.

#### 4.7.1. Adipogenic Differentiation

A total of 7000 cells were seeded on the slides in the 6-well plate. After 24 h, the media was changed to adipogenic medium. To confirm adipogenic differentiation, after 16 days, the cells were fixed with 4% paraformaldehyde (PFA) for 30 min, washed, and stained with a solution of Oil Red O (Bioptica) for 20 min and hematoxylin (Bioptica) for 2 min. They were then washed with distilled water. Images were obtained using optical microscopy. 

#### 4.7.2. Chondrogenic Differentiation

1 × 10^6^ cells were seeded on the slides in the 24-well plate, and after 2 h, the media was changed to chondrogenic medium. To confirm chondrogenic differentiation, after 14 days, cells were fixed with 4% PFA for 30 min, and Alcian Blue 8GX (Sigma-Aldrich) was used to stain the extracellular matrix mucopolysaccharides and hematoxylin (Bioptica) for 2 min. The staining solution was prepared by dissolving 1% Alcian Blue 8GX in 0.1 N HCl. This solution was filtered and added to each culture well for 30 min, and the cells were then washed with distilled water. Images were obtained using optical microscopy.

#### 4.7.3. Osteogenic Differentiation

In total, 5000 cells were seeded on the slides in the 12-well plate. After 2 h, the media was changed to osteogenic medium. To confirm osteogenic differentiation, after 21 days, cells were fixed with 4% PFA for 30 min and incubated in 0.2% Alizarin Red S (Sigma-Aldrich) for 5 min and hematoxylin (Bioptica) for 2 min. Then, they were washed with PBS (Gibco), and images were obtained using optical microscopy.

The cells of the control group were cultured with the ASC complete medium (Dulbecco’s Modified Eagle Medium (DMEM), 10% FBS, and 1% penicillin/streptomycin) and the cells were stained with hematoxylin to highlight the nucleus.

### 4.8. Statistical Analysis

Data were expressed as the mean ± standard deviation. Unpaired sample student’s *t*-tests were performed and differences between two groups were considered statistically significant, when *p*-value < 0.05.

### 4.9. Ethics Statement

The study respects all ethical requirements in its objectives and methodologies. We have strictly complied with widely recognized international codes of practice, such as the Nuremberg code, the Helsinki agreement, the conventions of the Council of Europe on human rights and biomedicine, with particular attention to EU legislation: 2001/83/EC, 86/609/EEC, and FP7 Decision nr 1982/2006EC. Human biological samples were required because we needed to test human cells, which have unique biological characteristics, distinct from those of animals. The overall intention of the project was to reduce the number of animal experiments. Only adult patients who were able to give consent were included. All the patients, which were the subjects of our study, gave their consent with regards to scientific treatment and publication of their clinic situation and images. We obtained written informed consent from all patients. This study was approved by our Internal Ethical Committee (CERM committee – 2019/27/Rig1), without any registration in public registry, because this study is not a clinical trial.

## 5. Conclusions

Although the enzymatic method, which has been used for 40 years in the laboratory in order to isolate cells, is the best available method, it is definitely not compatible with clinics, due to the long-lasting procedure and legal restrictions. Many efforts have been made to establish a mechanical method with a yield comparable to that of collagenase. Unfortunately, so far, none of them have displayed the same performance. In addition, in order to use it in vivo, a closed device is needed, and the method has to be fast, safe, standardized, and autologous. Rigenera^®^ addresses all of these requirements. In this study, we characterized in vitro the Rigenera^®^ product, focusing on two sites of adipose tissue harvesting. At first, we optimized the Rigenera^®^ operating time, demonstrating that 60 s of treatments allows a higher cellular yield to be obtained, in terms of the cell number and growth rate. This result optimizes the mechanical disaggregation and it can increase the clinical efficiency of the final product. Comparing the thigh and abdomen, our results showed that the thigh provides a higher number of mesenchymal-like cells, with a faster replication rate and a higher ability to form colonies. Finally, the immunophenotypic analysis confirmed that a much higher yield of ASCs was obtained from thigh samples. We can conclude that, by collecting adipose tissue from the thigh and treating it with the Rigenera^®^ device for 60 s, it is possible to obtain the most efficient product. This should lead surgeons to prefer the thigh as a harvesting site, especially for surgeries that require a small amount of injectable volume.

Our future work will involve a better characterization, conducted through a biomolecular analysis of the final product of Rigenera^®^, such as the expression level of genes involved in stemness; adipogenic, osteogenic, and chondrogenic differentiation or angiogenesis; inflammation; and cell aging.
